# Severe Cutaneous Adverse Reaction to Piperacillin-Tazobactam: A Case of Stevens-Johnson Syndrome

**DOI:** 10.7759/cureus.42839

**Published:** 2023-08-02

**Authors:** Hansraj Kumar, Alok Kumar, Subodh Kumar

**Affiliations:** 1 Pharmacology and Therapeutics, AIIMS Deoghar, Deoghar, IND; 2 Pathology and Laboratory Medicine, Shahid Nirmal Mahto Medical College Hospital (SNMMCH), Dhanbad, IND; 3 Pharmacology, AIIMS Deoghar, Deoghar, IND

**Keywords:** stevens-johnson syndrome, piperacillin-tazobactam, drug reaction, intravenous antibiotics, acute appendicitis

## Abstract

Stevens-Johnson syndrome (SJS) is a rare condition characterized by an exaggerated immune system response to triggers such as infections or drugs. It is characterized by blistering and exfoliation of the skin and affects mucosal surfaces, including the eyes, buccal cavity, and genitals. We report a case of a 50-year-old male who developed symptoms of SJS following a recent hospital admission for acute appendicitis The patient presented with fever, erythematous patches on the palms, abdomen, groins, and oral mucosa. The onset of symptoms occurred approximately four days after discharge from the hospital, where the patient had received treatment including intravenous antibiotics (Piperacillin-Tazobactam), ranitidine, tramadol, and intravenous fluids. He was diagnosed with SJS based on clinical and histopathological findings and was treated with supportive care and corticosteroids. He recovered after one week of hospitalization. This case highlights the importance of recognizing the potential risk of developing SJS following drug administration and the need for prompt identification and management of the condition to prevent complications and improve patient outcomes.

## Introduction

Stevens-Johnson syndrome (SJS) is a rare condition characterized by an overreaction of the immune system, triggered either by infections, commonly observed in children, or drugs, commonly seen in adults. SJS manifests with blistering and exfoliation of the skin, involving mucosal surfaces such as the eyes, buccal cavity, and genitals, although it is limited to 10% of the body’s total surface area in most cases [1]. Patients typically experience fever- and flu-like symptoms within one to three weeks of exposure to the causative drug [2]. Several drugs have been implicated in SJS, including allopurinol, piroxicam, ibuprofen, naproxen sodium, carbamazepine, lamotrigine, phenytoin, phenobarbital, sulfonamides, penicillin, cephalosporins, tetracyclines, and even radiation therapy [3]. Although a clinical diagnosis is typically made by the sign and symptoms itself, a skin biopsy is often required for confirmation.

## Case presentation

**Figure 1:**
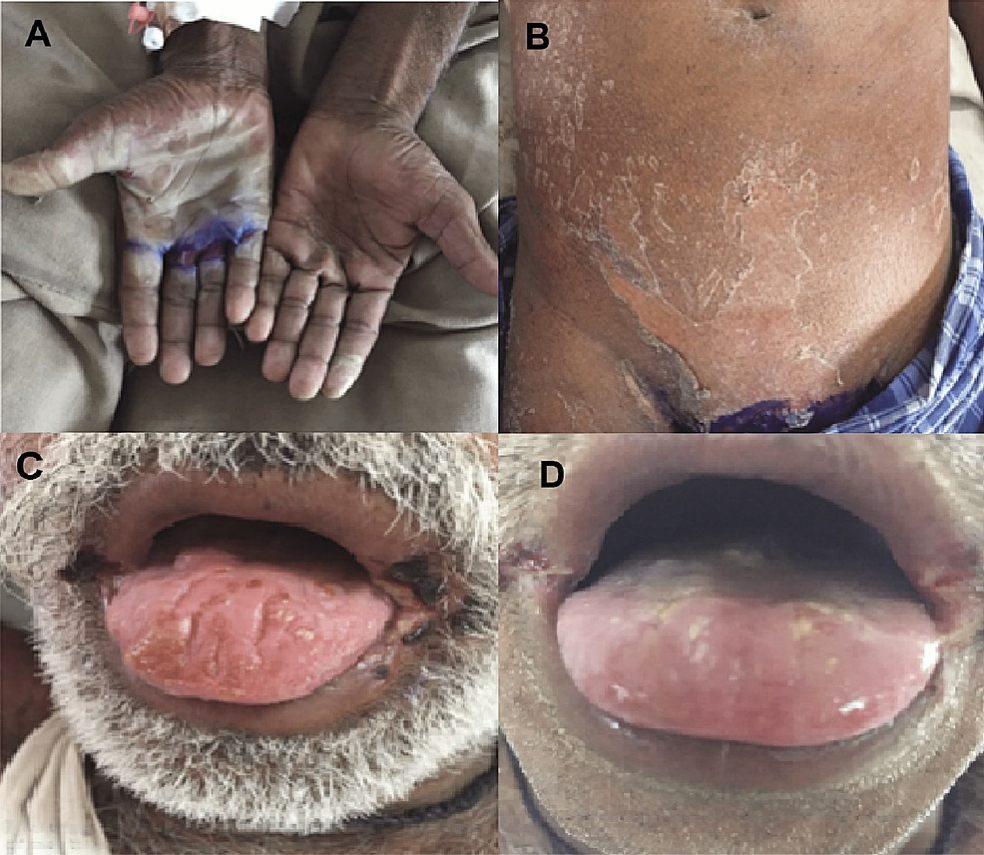
Exfoliation of palmar (A) and abdominal skin (B). Blistering of oral mucocutaneous junction at the angle of mouth and involvement of tongue (C) and its subsequent healing (D).

A 50-year-old male presented to the dermatology department with a three-day history of fever and erythematous patches over his palms, abdomen, and groin (Figures 1A-1C). The patient had recently been admitted to a local hospital for acute abdominal pain, which was diagnosed as acute appendicitis, and managed conservatively with intravenous piperacillin-tazobactam, intravenous ranitidine, intramuscular tramadol, and intravenous fluids. Four days after discharge, the patient began experiencing the aforementioned symptoms and sought care at our hospital.

The patient had no history of diabetes mellitus, hypertension, asthma, or previous allergic reactions to any drugs. A physical examination revealed erythematous mucocutaneous lesions around the angle of the mouth, lips, and tongue (Figure 1D), as well as multiple purpuric spots over the palms, abdomen, and genital region. During the patient’s hospital stay, they exhibited peeling of the skin from the palms, abdomen, and genital areas.

**Figure 2:**
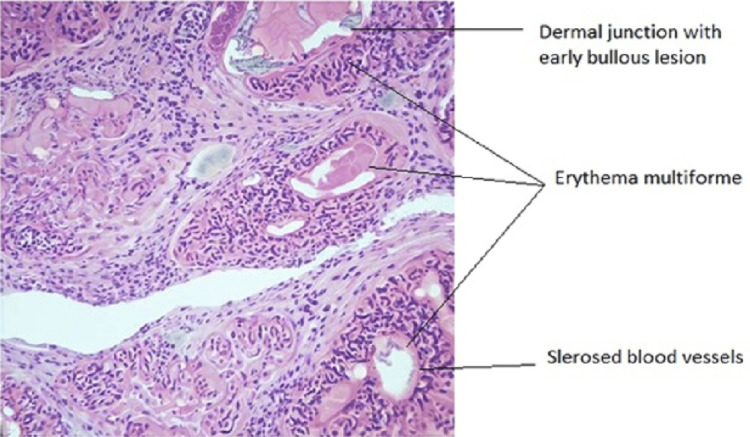
Histopathology evaluation of skin biopsy of the lesion

Blood Investigations showed mild leukocytosis, neutrophilia, and hypoproteinemia. Based on the clinical findings and the histopathology of the skin biopsy (Figure 2), a diagnosis of drug-induced SJS was made, with the suspected offender being the piperacillin-tazobactam combination.

We managed the patient conservatively with intravenous dexamethasone for two days, followed by oral prednisolone, intravenous pheniramine, intravenous cefoperazone, calamine lotion for dry areas, gentian violet paint for oozing areas, paracetamol tablets, and intravenous fluids. Gradually, the lesions started to disappear after seven days of conservative management (Figure 1D), and the patient was discharged with a tapering dose of oral prednisolone. During a follow-up visit to the dermatology outpatient department, the patient exhibited a complete resolution of symptoms without any sequelae after two weeks. The patient scored +6 on Naranjo’s causality scale, indicating a probable causal relationship between SJS and treatment with the piperacillin-tazobactam combination. The total score on the Naranjo scale was based on the following criteria: +1 for a previous conclusive report on this reaction and improvement of adverse events when the drug was discontinued each, and +2 for appearance of adverse event after the administration of suspected drug and no alternative causes that could on their own have caused the reaction, making a total score of +6 [4]. Similarly, the World Health Organization-Uppsala Monitoring Centre (WHO-UMC) system also indicated a “probable” association.

## Discussion

Piperacillin-tazobactam is an effective antibiotic against a broad spectrum of pathogens, including gram-positive and gram-negative aerobes, as well as anaerobes. It is commonly used for the treatment of nosocomial pneumonia, skin and soft-tissue infections, intra-abdominal abscesses, peritonitis, and as a postoperative prophylaxis. Although generally well tolerated, adverse reactions to piperacillin-tazobactam may include diarrhea (most common), swelling of the face and lips, headaches, and cutaneous reactions such as rashes, pruritus, and erythema multiforme. However, serious side effects are rarely reported [5].

SJS is a severe cutaneous hypersensitivity reaction that involves the separation of the epidermis from the dermis [6]. Commonly associated with sulfa drugs, anti-epileptics, and antibiotics, SJS is considered a medical emergency and necessitates hospitalization [7]. It typically presents with fever, sore throat, and fatigue, and involves mucocutaneous junctions, which almost always include the mouth and lips [8]. The lesions spread rapidly, leading to necrosis and sloughing of the skin. Clinical diagnosis is usually made based on characteristic signs and symptoms. SJS management focuses on identifying and eliminating the underlying cause, controlling symptoms, and minimizing complications. Recovery from SJS is generally slow and can take three to six weeks, depending on the extent, severity, and presence of complications. Compared to ranitidine or morphine use, more cases of SJS were linked with Piperacillin-tazobactam use. In this case, we did not carry out a re-challenge due to ethical considerations.

## Conclusions

The combination of piperacillin-tazobactam is a frequently used antibiotic for the treatment of various infections. Although it is generally well tolerated, it is crucial for physicians to be aware of the potential for serious adverse reactions such as SJS. This case serves as valuable knowledge for physicians, providing a better understanding of this specific adverse drug reaction associated with the use of piperacillin-tazobactam. By being informed about this potential side effect, healthcare professionals can take prompt action in identifying and managing SJS, thereby improving patient outcomes and safety.
